# Effective management of lung spindle cell carcinoma with ipilimumab, nivolumab, carboplatin, and paclitaxel, demonstrating efficacy in brain metastases treatment

**DOI:** 10.1002/rcr2.1366

**Published:** 2024-05-07

**Authors:** Susumu Noguchi, Atsushi Okamoto, Jun Nohara, Manabu Ishitoko, Toshiki Watanabe, Takaya Nakamura

**Affiliations:** ^1^ Respiratory Medicine Shiga General Hospital Moriyama Japan

**Keywords:** brain metastasis, ipilimumab, lung cancer, nivolumab, spindle cell carcinoma

## Abstract

A 76‐year‐old woman with cT1bN2M1b stage IVA spindle cell carcinoma of the right lower lobe of the lung, no driver gene mutation, and programmed death ligand 1 < 1%, was started on ipilimumab+nivolumab+carboplatin+paclitaxel. After two courses, the patient initiated maintenance therapy with ipilimumab+nivolumab. New multiple brain metastases were observed during treatment but resolved with continued treatment. We report a unique case of spindle cell carcinoma treated with ipilimumab+nivolumab+carboplatin+paclitaxel that resulted in long‐term response and resolution of new brain metastasis.

## Graphical abstract

We experienced a case of long‐term response to ipilimumab+nivolumab+carboplatin+paclitaxel for spindle cell carcinoma. The patient also showed efficacy in brain metastasis.

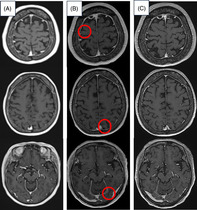

## INTRODUCTION

Spindle cell carcinoma is a subtype of sarcomatoid carcinoma estimated to account for approximately 13.3% of all sarcomatoid carcinomas. Its prognosis is poor because of its resistance to chemotherapy and radiotherapy.^1^ The prognosis of lung cancer has improved with the use of immune checkpoint inhibitors (ICIs).^2^ Ipilimumab is a fully human anti‐cytotoxic T‐lymphocyte antigen‐4 antibody, and nivolumab is a fully human anti‐programmed death (PD)‐1 antibody; the combination of both has been found to improve the prognosis of patients with lung cancer compared with conventional chemotherapy.^2^ In this report, we describe a case of spindle cell carcinoma of the lungs, including brain metastasis, that was treated with a combination of ipilimumab and nivolumab.

## CASE REPORT

A 76‐year‐old woman visited our hospital with suspected lung cancer due to a weight loss of 12 kg in 1 year and a nodule in the right lower lobe on computed tomography (CT). CT revealed a 17 mm nodule in the right lower lobe with enlarged inferior tracheobronchial lymph nodes. Endobronchial ultrasound‐guided transbronchial needle aspiration of these lymph nodes identified non‐small cell carcinoma, but yielded insufficient tissue for Oncomine Dx Target Test multi‐CDx system and PD‐L1 level assessments. Video‐assisted thoracic biopsy was performed for diagnostic purposes, revealing large spindle‐shaped cells that grew in bundles without epithelial‐like structures. Immunostaining for pan‐cytokeratin, desmin, p63, S100, and TTF‐1 was negative, leading to a spindle cell carcinoma diagnosis.

**FIGURE 1:**
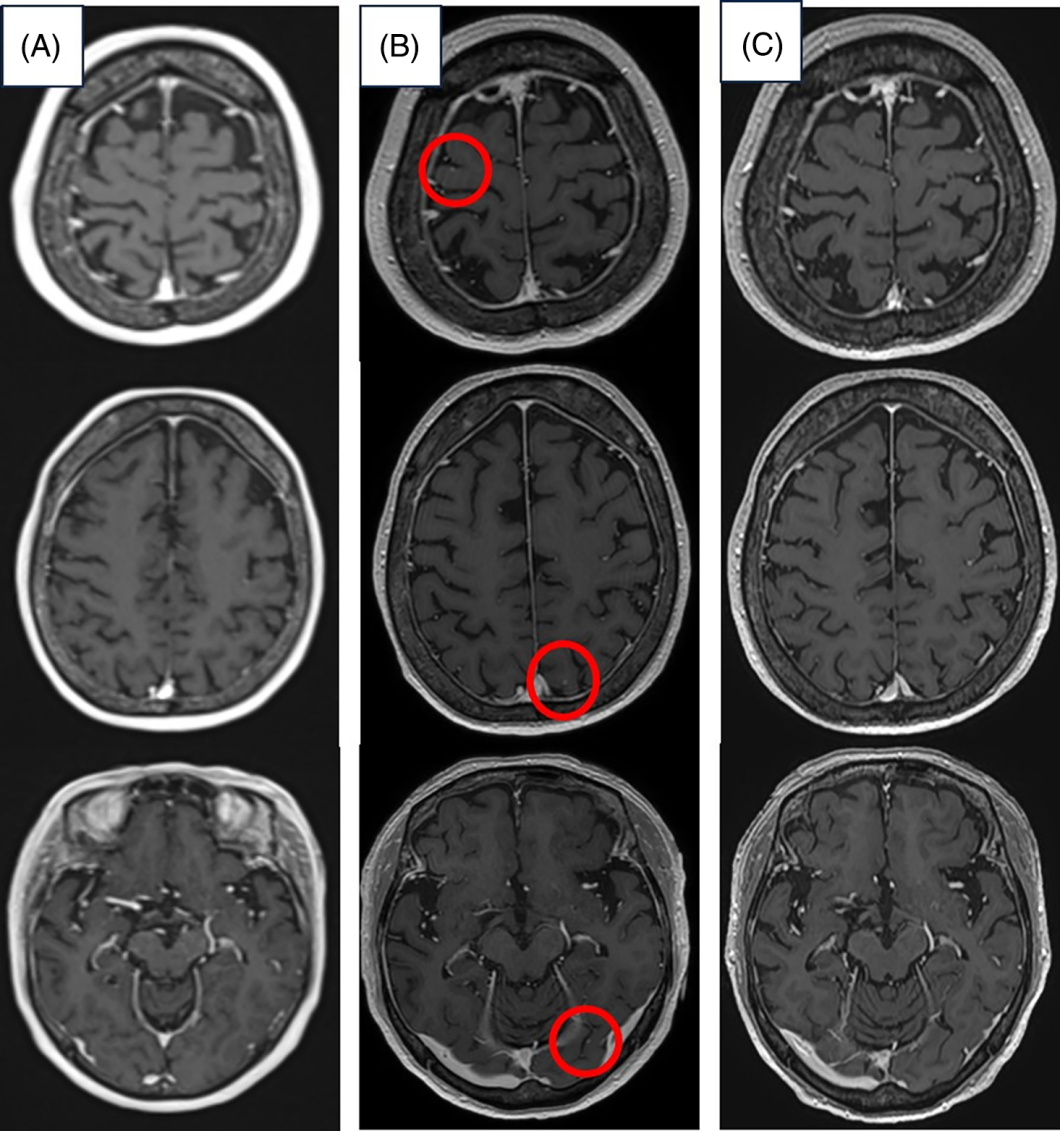
Progress of head MRI. (A) At the time of initial examination. (B) 7 months after the start of treatment. Micro‐nodules can be observed on both sides, which appeared to be brain metastases (red circles). (C) 10 months after the start of treatment, bilateral micronodules have disappeared.

**FIGURE 2:**
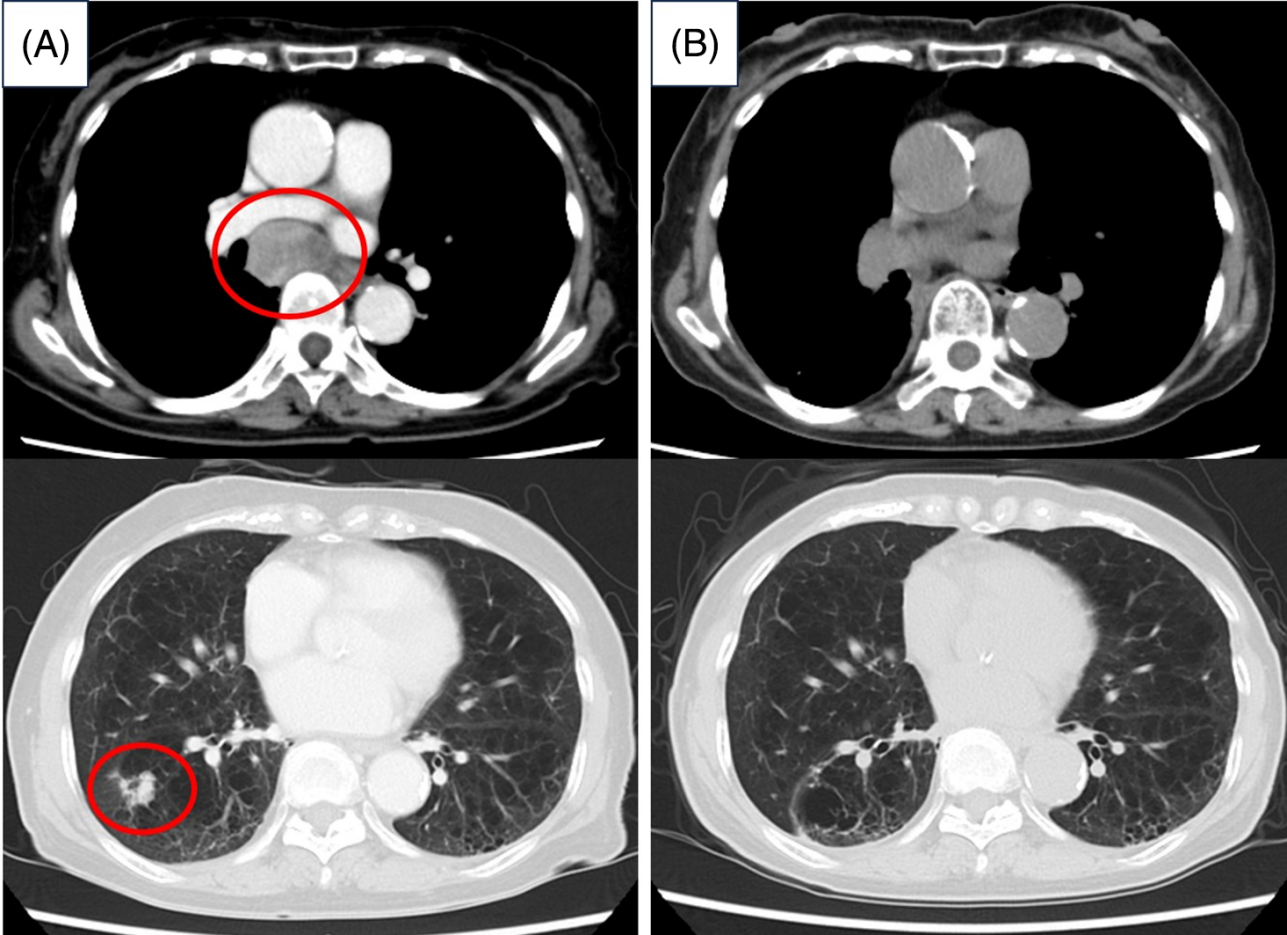
Progress of CT. (A) At the time of the initial examination. An enlarged mediastinal lymph node and a primary lesion in the right lower lobe are seen. (B) 1 year and 7 months after the start of treatment. Mediastinal lymph node shrinkage can be observed. The primary tumour is resected, with no evidence of recurrence.

Before initiating treatment, she underwent head magnetic resonance imaging (MRI) and positron emission tomography CT scans. These revealed no brain metastases; however, a right tarsal metastasis was observed. The diagnosis of cT1bN2M1b stage IVA was confirmed. The Oncomine Dx Target Test multi‐CDx system revealed mutations in the Kirsten rat sarcoma viral oncogene homologue *(KRAS) G12V*. Moreover, programmed death ligand 1 (PD‐L1) expression was <1%. Treatment with ipilimumab+nivolumab+carboplatin+paclitaxel was initiated in January 2021 as first‐line therapy. For the right tarsal metastasis, the patient was treated with 30 Gy of radiotherapy because of pain. Two courses of platinum‐based chemotherapy were administered, leading to a partial response; this was followed by ipilimumab+nivolumab (ipilimumab every 6 weeks, nivolumab every 3 weeks). Approximately 2 months after treatment initiation (day 73), grade 2 fever and diarrhoea were observed, which were considered immune‐related adverse events. Immunotherapy was discontinued, and treatment with prednisolone at 30 mg was started. The fever and diarrhoea were alleviated, but approximately 3 months later (day 100), thyroid‐stimulating hormone, free thyroid 4, adrenocorticotropic hormone (ACTH), and cortisol levels decreased. The patient was referred to an endocrinologist, who started levothyroxine and hydrocortisone after diagnosing hypothyroidism and isolated ACTH deficiency. Nivolumab therapy resumed about 2 months post‐interruption with improved adverse events, without recurrence elsewhere. However, post two nivolumab courses, a head MRI detected small nodules indicative of brain metastases. Preferring to avoid radiotherapy due to the absence of symptoms, the patient opted to continue nivolumab. Three months later, follow‐up MRI showed nodules had vanished (Figure 1), with no other recurrence, suggesting benefit from nivolumab monotherapy (Figure 2). The patient received 20 courses of nivolumab and continued the treatment.

## DISCUSSION

A patient with spindle cell carcinoma achieved long‐term response and brain metastases resolution after starting treatment with ipilimumab+nibvolumab+carboplatin+paclitaxel and continuing maintenance therapy. In non‐small cell lung cancer without driver mutations, combining ICIs with cytotoxic agents is advised. Both ICI combinations (ipilimumab+nivolumab) and the 9LA regimen (ipilimumab, nivolumab, plus cytotoxic agents) have been shown to extend overall survival (OS) over cytotoxic agents alone, irrespective of PD‐L1 status.^2^

Spindle cell carcinoma is resistant to chemotherapy and radiotherapy.^1^ However, ICIs are now available, and their efficacy has been reported. According to some reports, pembrolizumab alone is effective in treating lung spindle cell carcinoma.^1^ Notably, in a study involving 101 patients with either spindle cell carcinoma or giant cell carcinoma, a trend toward better OS with ICI was noted.^3^ However, these studies were based on populations dominated by patients with high PD‐L1 expression.

There are reports on the treatment of spindle cell carcinoma with ipilimumab+nivolumab; this regimen has been reported to be effective against renal mucinous tubular and spindle cell carcinoma.^4^ In this case, PD‐L1 expression was negative, and excluding first‐line treatment with a single ICI. Notably, no reports exist on using the use the ipilimumab+nivolumab regimen for lung spindle cell carcinoma. However, this combination proved effective regardless of PD‐L1 status, achieving a long‐term response, suggesting it could be a viable option for this condition.

Regarding brain metastases, preclinical data indicate that ICIs and activated T cells cross the blood–brain barrier, enabling ICIs to exert anti‐tumour effects in primary and metastatic brain tumours.^5^

Among trials that included patients with brain metastases, the Checkmate227 trial reported 5‐year survival rate of 20% in the group treated with ipilimumab+nivolumab and 6% in the group treated with chemotherapy alone, while the Checkmate9LA trial reported 3‐year survival rates of 28% and 12%,^2^ respectively; hence, its benefit for OS has been reported. In malignant melanoma, research indicates a 46% response rate to ipilimumab plus nivolumab for asymptomatic brain metastases and a 53.5% response rate with a 71.9% 3‐year survival rate in the CheckMate 204 trial, underscoring the treatment's efficacy for brain metastases. No reports suggest new lesions vanish with ICIs, but small, asymptomatic ones may improve, necessitating careful monitoring. Concomitant ipilimumab use can increase adverse events, including diarrhoea, fever, hypothyroidism, and ACTH deficiency, as seen in this case. Despite necessary caution, this evidence suggests ipilimumab+nivolumab+chemotherapy could be beneficial for spindle cell carcinoma and brain metastases treatment.

## AUTHOR CONTRIBUTIONS

Susumu Noguchi drafted the manuscript. All other authors critically reviewed, edited and approved final manuscript.

## CONFLICT OF INTEREST STATEMENT

None declared.

## ETHICS STATEMENT

The authors declare that appropriate written informed consent was obtained for the publication of this manuscript and accompanying images.

## Data Availability

The data that support the findings of this study are available from the corresponding author upon reasonable request.
